# No single, stable 3D representation can explain pointing biases in a spatial updating task

**DOI:** 10.1038/s41598-019-48379-8

**Published:** 2019-08-29

**Authors:** Jenny Vuong, Andrew W. Fitzgibbon, Andrew Glennerster

**Affiliations:** 1 University of Reading, School of Psychology and Clinical Language Sciences, Reading, RG6 6AL United Kingdom; 2 Microsoft, HoloLens, Cambridge, CB1 2FB United Kingdom

**Keywords:** Human behaviour, Visual system

## Abstract

People are able to keep track of objects as they navigate through space, even when objects are out of sight. This requires some kind of representation of the scene and of the observer’s location but the form this might take is debated. We tested the accuracy and reliability of observers’ estimates of the visual direction of previously-viewed targets. Participants viewed four objects from one location, with binocular vision and small head movements then, without any further sight of the targets, they walked to another location and pointed towards them. All conditions were tested in an immersive virtual environment and some were also carried out in a real scene. Participants made large, consistent pointing errors that are poorly explained by any stable 3D representation. Any explanation based on a 3D representation would have to posit a different layout of the remembered scene depending on the orientation of the obscuring wall at the moment the participant points. Our data show that the mechanisms for updating visual direction of unseen targets are not based on a stable 3D model of the scene, even a distorted one.

## Introduction

If a moving observer is to keep track of the location of objects they have seen earlier but which are currently out of view, they must store some kind of representation of the scene and update their location and orientation within that representation. There is no consensus on how this might be done in humans. One possibility is that the representation avoids using 3D coordinates and instead relies on a series of stored sensory states connected by actions (‘view-based’), as has been proposed in simple animals such as bees or wasps^1–3^, humans^4–6^ and deep neural networks^7,8^. Alternatively, the representation might be based on 3D coordinate frames that are stable in world-, body- or head-centered frames^9,10^, possibly based on ‘grid’ cells in entorhinal cortex^11,12^ or ‘place’ cells in the hippocampus^13,14^.

One key difference between these approaches is the extent to which the observer’s task is incorporated in the representation. For 3D coordinate-based representations of a scene, task is irrelevant and, by definition, the underlying representation remains constant however it is interrogated. Other representations do not have this constraint. Interestingly, in new approaches to visual scene representation using reinforcement learning and deep neural networks, the task and the environment are inextricably linked in the learned representation^7,15^. This task-dependency is one of the key determinants we explore in our experiment on spatial updating.

If the brain has access to a 3D model of the scene and the observer’s location in the same coordinate frame then, in theory, spatial updating is a straight-forward matter of geometry. It is harder to see how it could be done in a view-based framework. People can imagine what will happen when they move^16–18^ although they often do so with very large errors^19–24^. In this paper, we examine the accuracy and precision of pointing to targets that were viewed from one location and then not seen again as the observer walked to a new location to point, in order to test the hypothesis that a single 3D reconstruction of the scene, built up when the observer was initially inspecting the scene, can explain observers’ pointing directions. The task is similar to that described in many experiments on spatial updating such as indirect walking to a target^25–28^, a triangle completion task^29,30^, drawing a map of a studied environment including previously viewed objects’ location^19,31,32^, or viewing a set of objects on a table and then indicating the remembered location of the objects after walking round it or after the table has been rotated^33–36^. However, none of these studies have compared directly the predictions of a 3D reconstruction model with one that varies according to the location of the observer when they point, as we do here.

Spatial updating has been discussed in relation to both ‘egocentric’ and ‘allocentric’ representations of a scene^37–40^ and, in theory, either or both of these representations could be used in order to point at a target. An ‘egocentric’ model is assumed to encode local orientations and distances of objects relative to the observer^16,33,38,41^, while an allocentric model is world-based reflecting the fact that the relative orientation of objects in the representation would not be affected by the observer walking from one location to another^40^. People might use both^17,37,39^, and Wang and Spelke^38^ have emphasised consistency as a useful discriminator between the models. So, for example, disorientating a participant by spinning them on a chair should affect pointing errors to all objects by adding a constant bias if participants use an allocentric representation. The argument that Wang and Spelke^42^ make about disorientation conflates two separate issues, one about the origin and axes of a representation (such as ‘allocentric’ or ‘egocentric’) and the other about the internal consistency of a representation. In this paper, we focus on the latter. We ask whether a single consistent, but possibly distorted, 3D reconstruction of the scene could explain the way that people point to previously-viewed targets. In doing so, we concentrate on two potential sources of biases, namely errors in encoding (i) the location of the target boxes as seen from the start zone or maintaining this representation in memory (we lump these together) and (ii) the orientation and position of the observer as they walk. For any model based only on the 3D structure of the scene and the observer’s location within it, these are the two key elements that could contribute to bias in updating a target’s location during self-motion.

## Results

**Figure 1:**
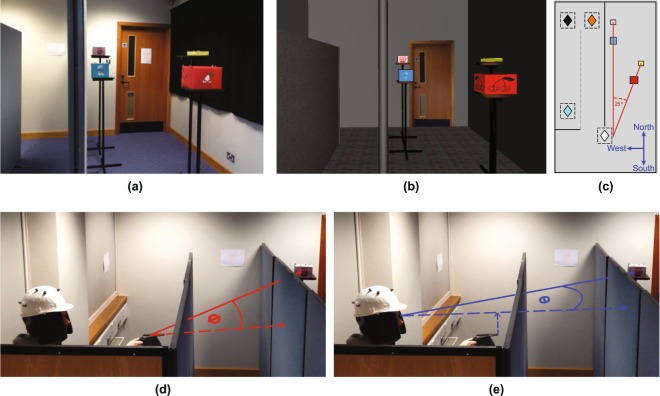
Experimental setup (**a**) Real world experiment and (**b**) in virtual reality (VR). The VR stimulus was created using in-house software written in C++. (**c**) Schematic plan view of one testing layout. Four boxes were arranged such that the blue and the pink, and the red and the yellow boxes lay along two separate visual directions as seen from the start zone (white diamond). From the start zone, the blue and the red box were always closer than the pink and the yellow box. The two visual directions subtended an angle of 25°. This angle was preserved for all box layouts (though distances varied, see Methods). Pointing to targets was tested at 3 different pointing zones (A = amber, B = black and C = cyan diamond). Black lines indicate positions of walls (in the real world, these were made from partitions). The dashed gray line indicates a wall that disappeared in VR after the participant left the start zone for the ‘direct’ condition. The icon in the corner shows the nominal ‘North’ direction. (**d**) We defined pointing direction as the direction indicated by the pointing device, labeled here as *ϕ*. (**e**) We did *not* use direction subtended at the cyclopean point (midpoint between the left and the right eye along the interocular axis) and the tip of the pointing device, labeled here as *θ*. Smurf image used with permission, copyright.

Our participants had access to a rich set of information about the spatial layout of four target objects whose position they would have to remember. In the real world or in a head mounted display, they had a binocular view of the scene and could move their head freely (typically, they moved ±25 cm). The targets consisted of four different colored boxes that were laid out on one side of the room at about eye height (see Fig. 1a) while, on the other side of the room, there were partitions (referred to as ‘walls’) that obscured the target objects from view once the participant had left the original viewing zone (‘Start zone’), see Fig. 1d,e. Most of the experiments were carried out in virtual reality (VR), see Fig. 1b, although for one experiment the scene was replicated in a real room (Fig. 1a). After viewing the scene, participants walked to one of a number of pointing zones (unknown in advance) where they pointed multiple times to each of the boxes in a specified order (randomized per trial, see Methods for details). Figure 1 shows the layout of the boxes in a real scene (Fig. 1a), a virtual scene (Fig. 1b) and in plan view (Fig. 1c, shown here for Experiment 1). The obscuring walls are shown on the left with a participant pointing in the real scene. The measure of pointing error used was the signed angle between the target and the ‘shooting direction’ (participants were asked to ‘shoot’ at the target boxes with the pointing device), as illustrated in Fig. 1d. Although not all participants had experience of shooting in video games or similar, the instruction was understood by all and this definition of pointing error gave rise to an unbiased distribution of errors for shots at a visible target (Supplementary Fig. S3), which was not the case for a considered alternative, namely the direction of the pointing device relative to the cyclopean eye (Fig. 1e). In the first experiment, the participant pointed to each of the target boxes eight times in a pseudo-random order (specified by the experimenter) from one of three pointing zones (shown in a, b and c respectively).

**Figure 2:**
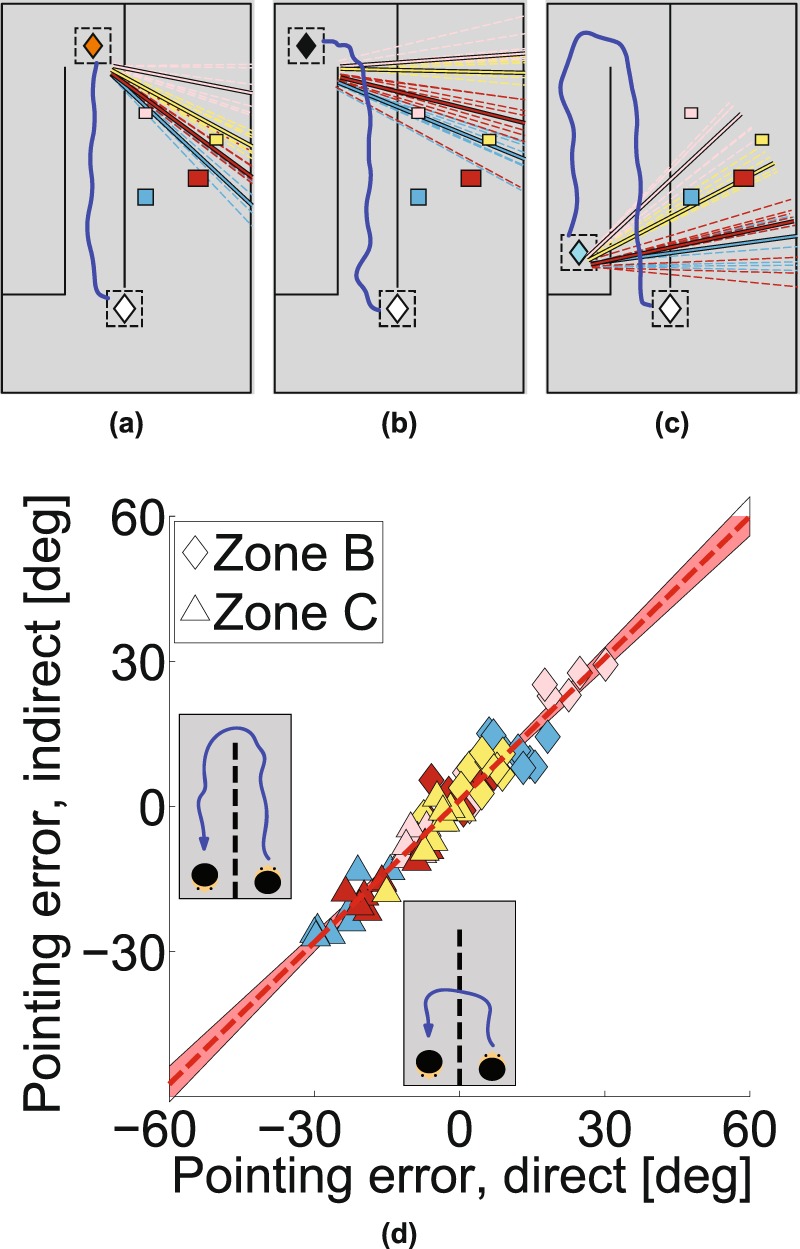
Pointing data from Experiment 1. Plan views in (**a**–**c**) show examples of pointing by one participant. The white diamond depicts the center of the start zone. The pointing zone is shown by (**a**) the amber diamond for zone A, (**b**) the black diamond for zone B and (**c**) the cyan diamond for zone C. The blue line shows the walking path from the start zone to the pointing zones. Pointing directions are colour coded according to the target; dashed lines show individual ‘shots’, solid lines indicate the mean for each target. (**d**) Pointing errors for direct and indirect walking paths to the pointing zones. Each symbol shows mean pointing error for 20 participants and for a given configuration of target boxes. Symbol colours indicate the target box, symbol shape indicates the pointing zone.

Figure 2a–c illustrate examples of the raw pointing directions in this condition for one participant. The participant makes large and consistent errors: For example, they point consistently to the ‘North’ of the targets from the northern pointing zones (A and B) but consistently to the ‘South’ of the targets from the southern zone, C (see Fig. 1c for definition of North). Not only this, some of the geometric features of the scene have been lost. For example, from zone C, the blue and yellow targets are almost co-aligned in reality but the participant points in very different directions to each. These features turn out to be highly repeatable, both across participants and in multiple versions of the task.

**Figure 3:**
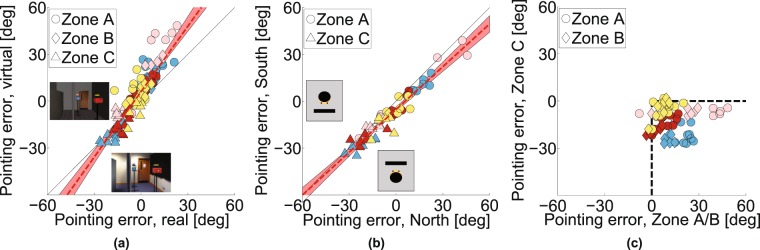
Pointing data from real world and different facing directions. (**a**) Experiment 1: Pointing errors from real world versus a virtual scene. (**b**) Experiment 2: Pointing errors for different initial facing directions. (**c**) Pointing errors for zone C compared to pointing errors for zone A and B (data from Experiment 1 and 2 combined).

Figure 2d shows the pointing errors gathered in two separate conditions plotted against each other (*Experiment 1*). The data shown are the mean pointing errors for 20 participants shown per target box (symbol color) for different box layouts and for different pointing zones (symbol shape). Despite the fact that the errors are substantial (from approximately −30° to +30°), there is a remarkable correlation between the errors in the two experiments (correlation coefficient 0.92, *p* < 0.001, slope 0.98; for individual participant data, see Supplementary Fig. S2). The ordinate shows the data from an experiment in VR when the layout of the walls was as shown in Fig. 2a–c, so that participants had to walk a long indirect route in order to arrive at Zone C, whereas the abscissa shows pointing errors when the experiment was repeated with one of the walls removed (see Fig. 1c) so that participants could walk direct to each of the pointing zones. For zone C especially, this makes a dramatic difference to the path length to get to the pointing zone, so any theory that attributed the pointing errors to accumulation of errors in the estimation of the participant’s location would predict a difference between the data for these two experiments, especially for pointing from Zone C but that is not the case. The data from all the other experiments reported in this paper conform to the same pattern, amounting to nine independent replications. We will use the data from this first experiment to build a simple model that predicts the pointing directions in all nine replications and test this model against the alternative hypothesis that the visual system uses a 3D model to generate pointing directions to the unseen targets. For example, Fig. 3a shows that there was also a high correlation between pointing errors when participants repeated exactly the same conditions in a real or a virtual environment (correlation coefficient 0.88, *p* < 0.001), although the range of pointing errors was greater in the virtual room (slope 1.42). Waller and colleagues^43^ also found a close match between performance in real and matched virtual environments.

**Figure 4:**
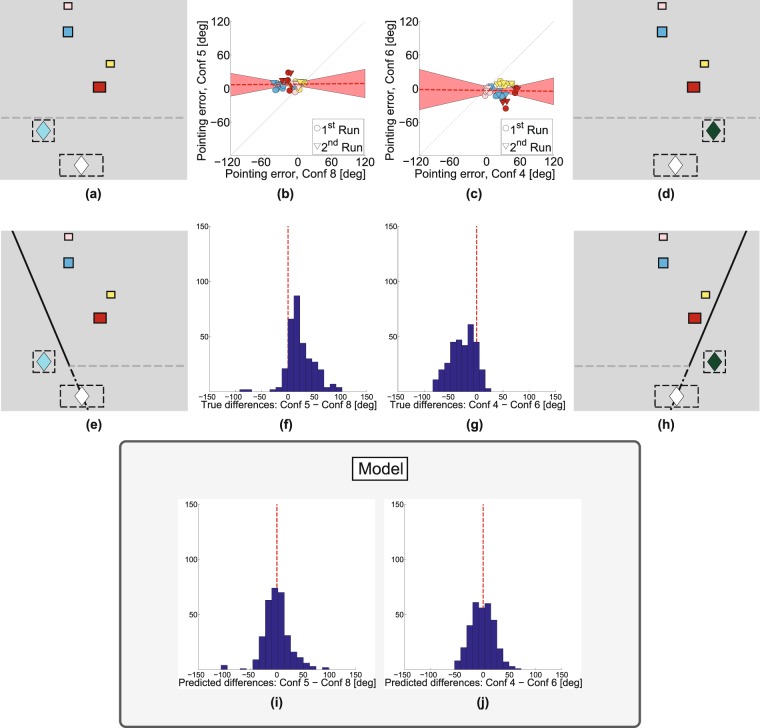
Results of Experiment 4. The same box positions have been tested at the same pointing zones, but differed in the wall orientation (**a**) versus (**e**) or (d) versus (**h**). Dashed gray lines indicate walls that appeared after the participant left the start zone. (**b**) Pointing errors are plotted for all the paired conditions with an ‘East-West’ wall versus the corresponding condition with a slanted ‘North-West’ wall (pointing from at zone C). (**c**) Similar comparison but now for a ‘North-East’ wall and pointing from zone E (emerald diamond). (**f**) Histogram of pointing differences between paired trials for the two different wall orientations pointing from zone C. (**g**) Same for zone E. (**i**),(**j**) show the same as (**f**),(**g**) respectively, except that they plot pointing differences with respect to the projection-plane model rather than with respect to ground truth. See Fig S1 for numbered list of scene configurations.

**Figure 5:**
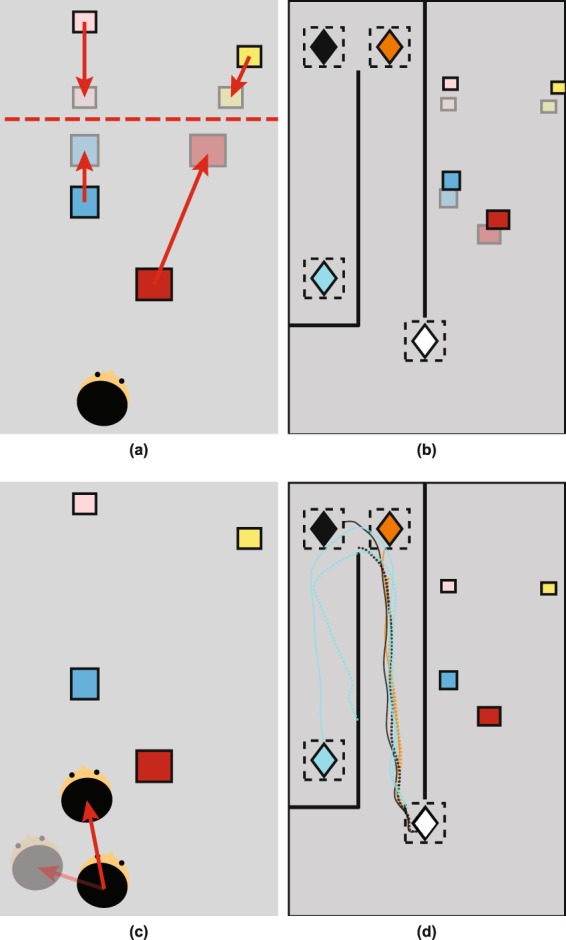
Schematic of abathic-distance-distortion and noisy-path-integration models (**a**) The abathic distance is shown by the dashed line and arrows show contraction towards this plane. (**b**) Example of target box locations shifted under the abathic distance model (opaque rectangles show true box positions). (**c**) Opaque head shows the observer’s true translation, the faded head shows the assumed translation under the noisy-path-integration model. Free parameters control errors in the estimate of translation and orientation on every step. (**d**) Solid lines show the walking paths of a participant, dotted lines show the misestimated walking path using this model.

We found the same pattern of pointing biases in a separate experiment that tested the role of egocentric orientation relative to the targets (*Experiment 2*). Figure 3b shows a high correlation between pointing errors when participants looked either ‘North’ or ‘South’ in order to view the image that told them which was the next pointing target. In these two versions of the experiment, participants’ rotation to point at the target was quite different, so any model based on egocentric direction at the moment the target was defined would predict a difference, but there was no systematic effect on the pointing errors (correlation coefficient 0.93, *p* < 0.001, slope 0.91). This high repeatability in the face of stimulus changes should be contrasted with the dramatic effect of changing the pointing zone. Figure 3c shows that, combining data across Experiments 1 and 2, pointing errors when participants were in zones A and B (Fig. 2a,b) were reliably *anticlockwise* relative to the true target location (positive in Fig. 3c, *M* = 12.1, *SD* = 20.1, *t*(949) = 18.7, *p* < 0.001 in a two-tailed t-test) while, when participants were in zone C (Fig. 2c), the errors were reliably *clockwise* (negative in Fig. 3c, *M* = −12.9, *SD* = 14.7, *t*(479) = −19.2, *p* < 0.001). *Experiment 3*, using pointing zones that were both to the ‘West’ and the ‘East’ of the target boxes showed a similar pattern of biases (see Supplementary Fig. S5). Another way to summarize the results is that, wherever the pointing zone was, the participants’ pointing directions were somewhere between the true direction of the target and a direction orthogonal to the obscuring wall. Expressed in this way, it is clear that the pointing zone itself may not be the key variable. Rather, it could be the spatial relationship between the target, the screen and the observer at the moment the participant points. In the next experiment, we examined paired conditions in which we kept everything else constant (box layout, start zone and pointing zone) other than the orientation of the obscuring wall.

**Figure 6:**
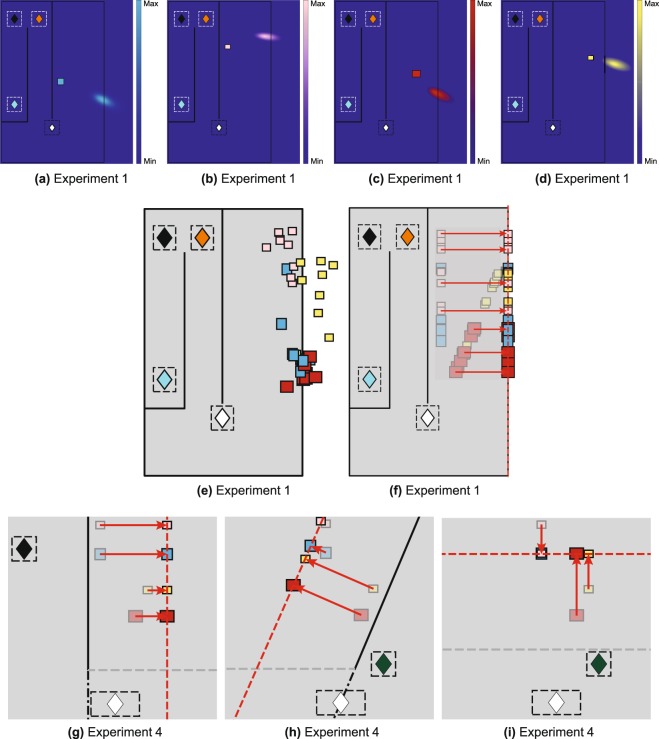
Retrofit target locations and the inspiration for the projection plane ‘model’. (**a**–**d**) Using all the pointing directions of all participants from three pointing zones it is possible to compute the most likely location of a target that would generate this pointing directions. Likelihood is plotted at every location and its peak is visible at the maximum-likelihood location. (**e**) Plan view of the maximum-likelihood positions of all boxes in each layout. Most of these locations are shifted towards a plane that is parallel to the central obscuring wall. (**f**) The projection plane model assumes that the projection plane is parallel to the wall, with a set distance (1.77 m), derived from the data in *Experiment 1*. (**g**–**i**) Illustrations of the projection-plane model applied to different configurations in *Experiment 4*.

The results of this experiment (*Experiment* 4) are shown in Fig. 4. Examples of paired stimuli are shown in Fig. 4a,e or Fig. 4d,h which show the same viewing zone (white diamond), the same pointing zone (cyan or green diamond) and the same box layout but a different obscuring wall (‘East-West’ versus slanted) which appeared after the participant left the viewing zone. It is clear that pointing zone alone is not the only determinant of the biases. Figure 4b,c plot the pointing error for each condition against the pointing error for its paired stimulus (i.e. changing only the wall orientation) and, unlike the conditions in Figs. 2 and 3, the data no longer lie close to the line of unity. Instead, as shown in Fig. 4f,g, the means of the distribution of differences between paired conditions (i.e. matched by participant, pointing zone, box layout and target box so that the only difference is the orientation of the obscuring wall changes) are significantly different from zero (zone C: *M* = 23.5, *SD* = 26.2, *t*(319) = 16.0, *p* < 0.001, zone E: *M* = −27.1, *SD* = 23.8, *t*(319) = −20.3, *p* < 0.001) and shifted in opposite directions for Fig. 4f,g. This is what one would expect if participants tended to point somewhere between the true target direction and a direction orthogonal to the obscuring wall, just as they did in *Experiment 1* (Fig. 3c). We will return later to the model predictions shown in Fig. 4j,i.

## Models

We examined five types of model. First, participants may have remembered the layout of the scene correctly but mis-estimated their location when they pointed (Fig. 5c,d). Details of the models are given in Supplementary Information. For this **noisy path integration model**, the errors predicted by the model are larger for a longer than a shorter path (Supplementary Fig. S4) whereas this is not the case for participants (indirect versus direct path to zone C, Fig. 2d).

**Figure 7:**
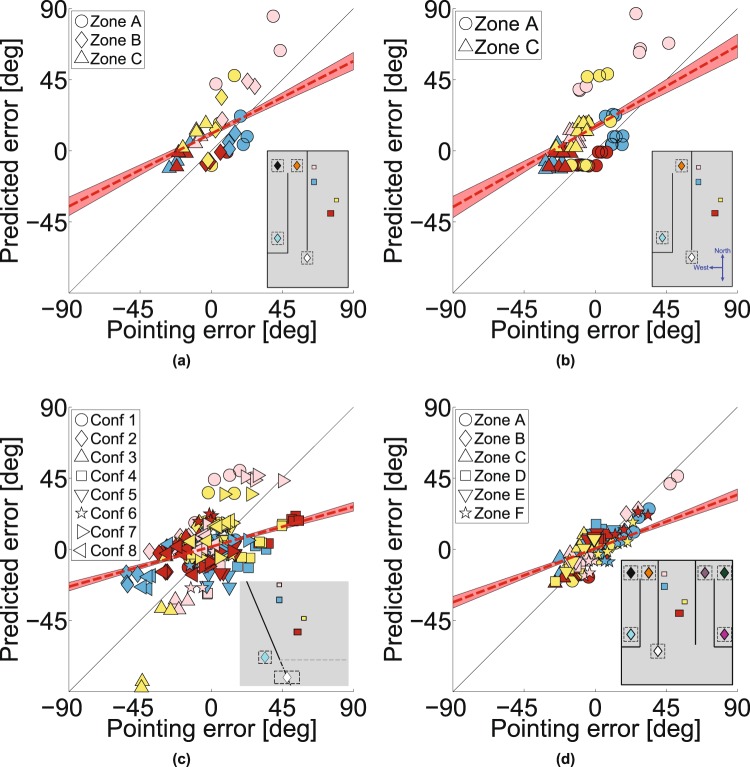
Predictions of the ‘retrofit’ model. For box layouts that were shown in Experiments 1, 2 and 4, a configuration of box locations was determined that would best explain the pointing data (see Supplementary Fig. S5). Predictions of the model are shown for (**a**) Experiment 1, (*R* = 0.52, *p* < 0.001, RMSE = 24.1, slope = 0.51), (**b**) Experiment 2, (*R* = 0.54, *p* < 0.001, RMSE = 29.3, slope = 0.57), and (**c**) Experiment 4 (*R* = 0.32 and *p* = 0.0014, RMSE = 34.1, slope = 0.28). Scene configurations are shown in Supplementary Fig. S1, (**d**) Experiment 3 was treated separately as the box layouts were different (see Supplementary Fig. S5) and all 6 pointing zones were used to predict box locations (*R* = 0.40 and *p* < 0.001, RMSE = 25.7, slope = 0.38).

Alternatively, participants may have mis-represented or mis-remembered the spatial structure of the target layout. We examined two variants on this hypothesis. First, we fitted the data assuming that participants generated a distorted spatial representation when they viewed the scene at the start zone. We used a well-established model of spatial distortions^44,45^ in which the distance of objects is judged correctly at an **‘abathic’** distance and there is a compression of visual space towards this plane as illustrated in Fig. 5a (details in Supplementary Information). A problem for this model, just like the noisy-path-integration model, is that it makes no prediction about the effect of the slant of the obscuring wall. The second variant of spatial distortion models that we applied to the data was much more extreme in that that allowed the location of the target boxes to vary freely to maximise the likelihood of the pointing data, i.e. **‘retrofitting’** the scene to match the data (Supplementary Information for details). As such, it is hardly fair to describe this a ‘model’ in the sense of having any predictive value since the 3D configuration of the supposed internal representation is determined entirely by the data. The result of doing this for *Experiment 1* is illustrated in Fig. 6a–d. We used the pointing directions of all 20 participants from 3 pointing zones per target box in each condition to calculate the most likely location of the box that would explain their pointing responses. Figure 6a shows the result of this calculation for the blue box in one condition. It is clear that the derived box location is far from the true location. Figure 6b–d show the same for the pink, red and yellow target boxes respectively (see Supplementary Fig. S5 for the same model applied to *Experiment* 3). Figure 6e marks the location of the peaks of the likelihood distributions illustrated in Fig. 6a–d and all other conditions in *Experiment 1*. These points tend to cluster around a plane that is parallel to the obscuring wall whereas the true locations of the target boxes (shown in translucent colors in Fig. 6f) are nothing like this.

**Figure 8:**
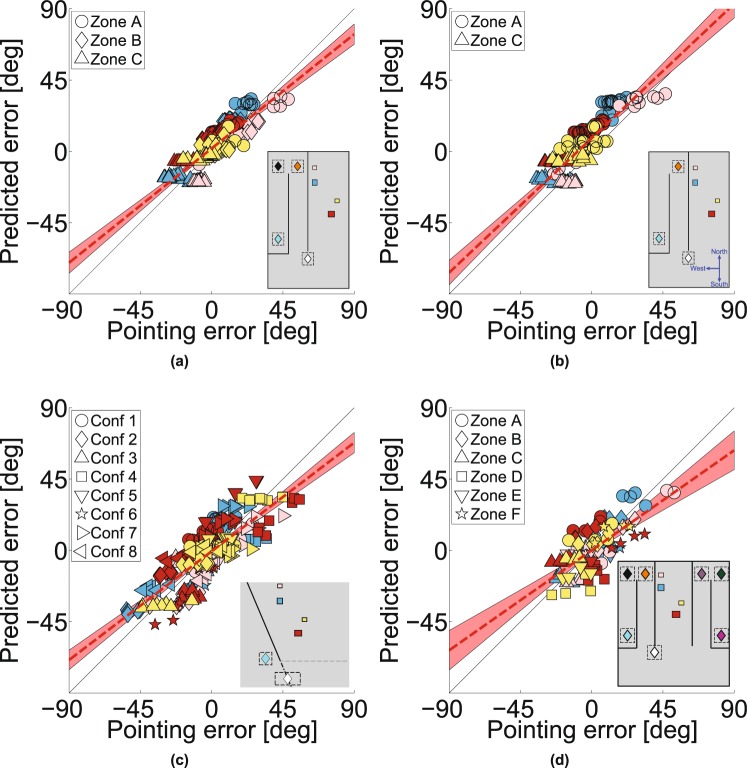
Predictions of the projection-plane model. As for Fig. 7 but now showing the pointing errors predicted by the projection-plane model. (**a**) Predictions for Experiment 1 (*R* = 0.86 and *p* < 0.001, RMSE = 8.6, slope = 0.80), (b) Experiment 2 (*R* = 0.86 and *p* < 0.001, RMSE = 12.2, slope = 0.89), (**c**) Experiment 4, (*R* = 0.73 and *p* < 0.001, RMSE = 12.2, slope = 0.73) and (**d**) Experiment 3 (*R* = 0.81 and *p* < 0.001, RMSE = 11.8, slope = 0.66).

The fourth type of model we tested, was inspired by the results of the retrofit model. In this sense, it is *post-hoc*, being based on the results for *Experiment 1*. Figure 6f, shows the zero-parameter model based on the retrofit target locations in Fig. 6e. According to this model, participants will point to remembered targets by assuming that they all lie on a single plane that is parallel to and 1.77m behind the central obscuring wall. The value of 1.77m is simply taken from the best fit to the points shown in Fig. 6e. Figure 6g–i show examples of this **projection-plane** rule applied in other conditions.

In the Discussion, we consider a different type of model that is based on a quite different proposal: Participants’ pointing directions lie between the correct response to a specific target and a ‘prototypical’ response to stimuli in the same **‘category’**^46,47^. Pointing direction might then be a weighted sum of the true direction of the target and its direction from other pointing zones. In *Experiment 1*, if pointing responses were a weighted sum of the true direction of the target and the direction of the target from another pointing zone (e.g. zone B when the participant was at zone C) then the result would be a pointing direction that was more orthogonal to the wall than the true direction of the target, as we found. The best-fitting weighting between zones to explain the data in *Experiment 1* is 0.82:0.18 (actual pointing zone:alternative zone) and, in this case, the RMSE of the data with respect to the model is only slightly worse than for the projection plane model. However, when more stimuli are mixed together, as in *Experiment 4*, these hypotheses diverge (see Discussion).

### Model comparison

Supplementary Table S1 shows a comparison of the fits of the noisy-path-integration model, the abathic-distance-distortion model and the projection-plane model for the data in *Experiment 1*. The RMS errors are lower overall for the projection-plane model and also for 17/20 of the participants. More important, as we have discussed already, the noisy-path-integration and abathic-distance-distortion models cannot account for the effect of the obscuring wall orientation (Fig. 4). However, given that the projection plane model was derived from the data in *Experiment 1*, a more critical test is to see how well it compares to other models in *Experiments 2* to *4* where it provides a zero-parameter prediction of the data.

The most stringent possible test is to compare performance of the projection-plane model against that of the ‘retrofit’ model. The latter is provided with all the data and asked to derive a 3D structure of the scene that would best account for participants’ pointing directions using a large number of free parameters (24) so, unlike the projection-plane model, it does not make predictions. Supplementary Table S2 shows a measure of goodness of fit of the projection plane model and the retrofit models, adjusted for the number of parameters in each (using Bayesian (BIC) and Akaike (AIC) Information Criteria). According to the BIC, the projection plane model outperforms the retrofit method in all 4 experiments. This can be seen graphically in Fig. 7, which shows the predicted pointing errors from the retrofit model plotted against the actual pointing errors, while Fig. 8 shows the same for the projection-plane model: the slopes are all steeper for the projection plane model.

Returning to effect of the obscuring wall, Fig. 4j,i show that the projection-plane model accounts for the bias that is caused by the orientation of the wall in that experiment. Using paired trials (i.e. with everything else kept the same except for the wall orientation), these plots show the distribution of differences in pointing directions relative to the *predicted* direction of the target. Unlike the original biased pointing directions (Fig. 4f,g), the mean of the distribution is no longer significantly different from zero (*M* = −1.81, *SD* = 22.0, *t*(319) = −1.48, *p* = 0.140), showing that the model has accounted well for the pointing biases.

## Discussion

For a spatial representation to be generated in one location and used in another, there must be some transformation of the representation, or its read-out, that takes account of the observer’s movement. The experiments described here show that in humans this process is highly inaccurate and the biases are remarkably consistent across participants and across many different conditions.

The data appear to rule out several important hypotheses. Crucially, any hypothesis that seeks to explain the errors in terms of a distorted internal model of the scene at the initial encoding stage will fail to capture the marked effects of the obscuring wall, which is generally not visible at the encoding stage (see Fig. 4). We examined standard models of a distorted visual world, namely compression of visual space around an ‘abathic’ distance^44,45^ (details in Supplementary Information) but even when we allow *any* type of distortion of the scene that the observer sees from the starting zone to explain the data (provided that the *same* distortion is used to explain a participant’s pointing direction from all pointing zones and any wall orientation), this type of model still provides a worse fit to the data than our projection-plane model (see Figs 7,8 and Supplementary Table S2). The post-hoc nature of the ‘retrofit’ model could be considered absurdly generous. The fact that it is *still* worse than a zero-parameter model generated from the first experiment provides strong evidence against 3D reconstruction models of this type.

We also examined and ruled out a hypothesis based on noisy path integration^48^ as an explanation of our data. One strong piece of evidence in relation to such hypotheses is the high correlation between the magnitude of errors participants make when they walk to a pointing location either via a long or a short route (Fig. 2d and Supplementary Fig. S2). These data suggest that it is not the route to the pointing zone that can explain the errors. Instead, it is something about the location and the scene when the participant gets there.

A non-geometric explanation of the biases was raised in Models, namely that participants point somewhere between the correct direction of the target and its direction in all the conditions encompassed within a particular ‘category’^46,47^. It is not obvious how such a category should be defined. For example, in Experiment 4 when 32 different conditions were randomly interleaved and all other factors were matched, we found that pointing biases depended systematically on the orientation of the wall (see Fig. 4). Since a random set of conditions preceded any given trial, a ‘prototypical’ category based on all previous trials could not account for a difference in pointing bias that is dependent on the orientation of the wall.

It has often been shown that the observer’s orientation can have a significant effect on pointing performance. For example, when the orientation of the participant in the test phase differs from their orientation during learning of the scene layout this can influence pointing directions (for both real walking^49^ or imaginary movement^17,50^ in the test phase). Röhrich *et al*.^32^ showed that a participant’s location when pointing can have an important effect on the reference frame they use to imagine the target. Meilinger *et al*.^51^ investigated the effect of adding walls to an environment and showed they have a significant effect. However, the authors did not examine the biasing effects of moving to different pointing locations nor can the results be compared directly to ours since they report ‘absolute errors’ in pointing, a measure that conflates variable errors and systematic biases. In fact, this is a common problem in the literature; many other papers report only absolute errors in pointing^17,19,21,22,24,52–54^.

‘Boundaries’, such as the walls in our experiment, have often been considered as important in determining the coding of spatial positions^55–57^ although these earlier papers do not provide predictions about the bias in representation of objects the other side of a wall. Opaque boundaries can have an effect on the way that hidden objects are categorised. For example, spatial memories can be biased towards the centres of enclosed regions^58^ (a type of categorical bias, as discussed above) but there are no enclosed regions in our experiment and so it is hard to see how this could explain the biases we report here. We include all the pointing data online (see Data Availability) with code to produce Fig. 8 so testing any well-specified alternative model should be straightforward.

The fact that the most lenient 3D reconstruction model does not do well in explaining our data raises the question of what participants do instead. The ‘projection-plane’ model is essentially a re-description of the data in one experiment, rather than an explanation, albeit one that then extends remarkably successfully to other situations (‘remarkably’ because it is a zero-free-parameter model). It might seem at first sight that the projection plane model implies that the brain must generate a 3D model of the scene, including a projection plane, but that is not the case. Recent neural network models show that it is possible to predict a novel view of a scene from a novel location (which is very close to our task)^8^. The network is able to do this given only one or two images of the novel scene and, crucially, millions of examples of moving through similar environments so that the network learns how images change when the camera moves. Explaining what it is that the network has achieved could be done using a plan view of the scene and the novel viewpoint, just as we use a plan view and a projection plane to illustrate our model, but in neither case is it necessary to assume that the network (or the brain) is actually using a 3D model. Indeed, for the neural network demonstration^8^, the whole point is that it is not. We are suggesting instead that the brain uses a heuristic and that systematic errors in human pointing reflect failures of that heuristic. The fact that there are quite different errors for different orientations of the wall strongly suggests that, as the observer walks, images of the obscuring wall are an important input to the updating process. Two factors in the data are relevant when considering how this heuristic might operate. First, when a participant is directly facing the obscuring wall, the model predicts that errors should be at a minimum, suggesting that this view may be treated as ‘canonical’ in some sense. This echoes the way that people interpret images on slanted surfaces as if they were viewing them ‘front on’^59^. Second, the fact that target objects are modelled as being on a plane suggests the updating process lacks much of the subtlety of a geometric calculation. There is increasing interest in non-metric models of various kinds that avoid 3D calculations^8,60–62^.

Finally, it is worth considering what effect such large biases might have in ordinary life. The most relevant data from our experiments in relation to this question are, arguably, the pointing biases that we recorded from the very first trial for each participant. We made sure that, on this first trial, the participant was unaware of the task they were about to be asked to do. Biases in this case were even larger than for the rest of the data (Supplementary Fig. S2). If these data reflect performance in daily living, one might ask why we so rarely encounter catastrophic consequences. One response is that the task we asked our participants to carry out is a relatively unusual one and, under most circumstances, it is likely that visual landmarks will help to refine direction judgments *en route* to a target.

Our conclusions are twofold. First, although human observers *can* point to remembered objects, and hence must update some form of internal representation while the objects are out of view, we have shown that they make highly repeatable errors when doing so. Second, the best explanation of our data is not consistent with a single stable 3D model (even a distorted one) of the target locations. This means that whatever the rules are for spatial updating in human observers, they must involve more than the structure of the remembered scene and geometric integration (even with errors) of the path taken by the observer.

## Methods

In VR, participants viewed the stimuli through a binocular head-mounted display NVIS SX111 with horizontal view of 102°, vertical view of 64° and binocular overlap of 50°. Each display for the right and the left eye had a resolution of 1280 × 1024 pixels refreshed at a rate of 60 Hz and an end-to-end latency of 2 frames. The head-mounted display was tracked at a rate of 240 Hz.

In the real environment, participants wore a tracked hardhat with blinkers on the side to imitate the horizontal field of vision of the head-mounted display. The participants were blindfolded when guided to the start zone by the experimenter. The target boxes had markers stuck to them so that they could be tracked and their position recorded during trials. In both virtual and real environments, participants used a hand-held, fully-tracked pointer resembling a gun, to indicate a pointing direction with the direction of the pointer and with a button press.

All participants had normal or corrected-to-normal vision and could distinguish the colors of the target boxes. Their acuity and stereoacuity were tested before the start of the experiment: all had 6/6 vision or better and normal stereopsis with 60 arc seconds or better.

### Informed consent

The study and all the experimental protocols received the approval of the University of Reading Research Ethics Committee. All methods were carried out in accordance with relevant guidelines and regulations. Informed consent for study participation and for publishing identifying information/images was obtained from all participants.

## Experiment 1

There were two parts to the experiment. In one, carried out in VR, on different trials participants walked via two different routes (‘direct’ and ‘indirect’) to get to the pointing zones from which they pointed to the remembered targets. In the other, participants carried out one of these tasks (‘indirect’ route) in a very similar environment in the real world. The very first trial the participants experienced was one in the real world.

### Participants

For the first experiment, we recruited 22 participants (aged 19–46) who were paid £10 per hour. Data from 2 participants had to be discarded as one failed to understand the task and one felt too uncomfortable wearing the head-mounted display. 19 out of the 20 were naïve to the experiment, and 1 was a researcher in our lab who had prior knowledge about the task (see Supplementary Table S1).

### Procedure

In VR, in order to start a trial, participants walked towards a large green cube with an arrow pointing towards it in an otherwise black space then, as soon as they stepped inside the cube, the stimulus appeared. In both real and virtual experiments, the participants then viewed 4 boxes from this start zone. They were told that from where they were standing, the blue box was always closer than the pink box and both were in the same visual direction. Likewise, the red box was always closer than the yellow box and, again, both were in the same visual direction when viewed from the start zone (see the example plan view in Fig. 1a–c). When they had finished memorizing the box layout, they walked behind a ‘wall’ (partition) towards a pointing zone. The layout of the partitions is shown in Fig. 1. The ‘inner’ north-south partition, shown by the dashed line in Fig. 1c, was removed in the ‘direct’ walking condition (only tested in VR). The participant had to point 8 times to each of the 4 boxes in a pseudo-random order, i.e. 32 pointing directions in all. The end of each trial was indicated by the whole scene disappearing in VR (replaced by the large green box) and in the real world the experimenter told the participant to wait to be guided back blindfolded to the start.

### Box layouts and stimulus

8 participants were tested on 9 box layouts (1,440 pointing directions in total in VR per participant: ‘Indirect’ — 9 layouts × 4 boxes × 3 zones × 8 shots, ‘Direct’ — 9 layouts × 4 boxes × 2 zones × 8 shots), whereas the remaining 12 participants were tested on 4 layouts (640 trials in VR per participant). All participants repeated the ‘indirect’ condition in the real world in exactly the same way as they did in VR. All the box layouts are shown online in an interactive website, see Data Availability, including the raw pointing data in each case. The virtual and the real stimulus were designed to be as similar as possible, with a similar scale, texture mapping taken from photographs in the laboratory and target boxes using the same colors and icons, see Fig. 1a,b.

The box positions in the 9 box layouts were chosen such that the following criteria were satisfied, see Fig. 1c:

- The blue and the pink boxes were positioned along one line (as seen in plan view) while the red and the yellow box were positioned along a second line. The two lines intersected inside the start zone.
- All box layouts preserved the box order: this meant that the blue box was always in front of the pink box, and the red box was in front of the yellow box, but the distances to each box varied from layout to layout.
- The 2 lines were 25° apart from one another. This number allowed a range of target distances along these line while the boxes all remained within the real room.

## Experiment 2

This experiment aimed to identify the influence of the facing orientation at the pointing zones. 7 participants, all of whom had been participants in the first experiment, viewed the same 6 layouts as in *Experiment 1* but in VR only. They then walked to either zone A or zone C, stopped and faced a poster located either to the ‘North’, ‘South’, or ‘West’ of the pointing zone (unlike *Experiment 1* when there was only one poster position per pointing zone, see Supplementary Fig. S1. Otherwise, the procedure was identical to *Experiment 1*, with the exception that the participant pointed 6 times (rather than 8) to each of the boxes in a random order at the pointing zones. Data are shown in Supplementary Fig. S2 and Fig. 3b.

## Experiment 3

The third experiment was also carried out only in VR and here the testing room was scaled (in the x-y plane) by a factor of 1.5, i.e. the height of the room did not change. The pointing zones A, B and C were mirrored along the center line of the room to create an additional 3 pointing zones D, E and F, see Supplementary Fig. S1 but the physical size of the laboratory dictated that participants could only go to one side of the scene or the other (either zones A, B, C or zones D, E, F). The participants did not know whether they would walk to the left or to the right from the start zone until the moment that they pressed the button (when the boxes disappeared). There were 6 participants, 3 of whom had taken part in the previous experiments.

## Experiment 4

The purpose of this experiment was to compare conditions in which the start zone, the pointing zone and the box layout were matched but the orientation of the wall was different. The scene layouts for this experiment are shown in Supplementary Fig. S1. There were 4 pointing zones B, C, D and E and 4 box layouts. Ten participants were recruited for this study (6 of whom had taken part in earlier experiments in the study). The experiment was carried out in VR. The procedure for this experiment was similar to *Experiments 1*–*3* with the following differences: After viewing the target boxes from the start zone and pressing a button, a 1.8m tall wall appeared in front of the participant obscuring the targets (gray dotted line in Supplementary Fig. S1). When the participant left the start zone, another wall appeared so that there was only a single wall obscuring the targets from that moment on (see black solid line in Supplementary Fig. S1). A green square on the floor appeared at the pointing zone and a poster at eye height at the pointing zone appeared indicating which target box they should shoot at (6 times to each of the 4 boxes in a pseudo-random order for one trial). One run consisted of 32 trials (8 conditions shown in Supplementary Fig. S1 times 4 box layouts) presented in random order. Each participant carried out two runs (1,536 pointing directions).

## Supplementary information

LaTeX Supplementary File

Supplementary Information for Vuong, Fitzgibbon and Glennerster (2019)

## Data Availability

The datasets generated during and/or analysed during the current study are available in the github repository, https://github.com/jnyvng/PointingData. Code to reproduce an example figure (Fig. 8) can also be found in the repository. Also, there is an interactive website showing the raw pointing data for all the experiments: http://www.jennyvuong.net/dataWebsite/.
