# Supplementary figures and images for: No single, stable 3D representation can explain pointing biases in a spatial updating task
Source: Sci Rep. 2019 Aug 29;9:12578. doi: 10.1038/s41598-019-48379-8 (PMC6715735; doi:10.1038/s41598-019-48379-8)

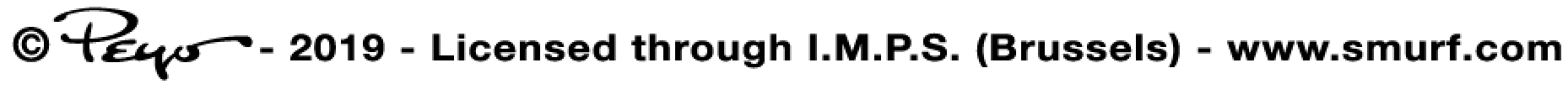

Supplement: Supplementary file 1 — LaTeX Supplementary File [file 41598_2019_48379_MOESM1_ESM.png]
