# Supplementary material for: No single, stable 3D representation can explain pointing biases in a spatial updating task
Source: Sci Rep. 2019 Aug 29;9:12578. doi: 10.1038/s41598-019-48379-8 (PMC6715735; doi:10.1038/s41598-019-48379-8)
Supplement: Supplementary file 2 — Supplementary Information for Vuong, Fitzgibbon and Glennerster (2019) [file 41598_2019_48379_MOESM2_ESM.pdf]

No single, stable 3D representation can explain pointing biases in a spatial updating task

Jenny Vuong[1,*], **Andrew W. Fitzgibbon**[2], **and Andrew Glennerster**[1,*]

[1]University of Reading, School of Psychology and Clinical Language Sciences, Reading, RG6 6AL, United Kingdom
[2]Microsoft, HoloLens, Cambridge, CB1 2FB, United Kingdom
[*]me@jennyvuong.net (JV), a.glennerster@reading.ac.uk (AG)

ABSTRACT

Supplementary Information

Virtual Reality

The tracking system comprised 14 MX3/T20/T20-S Vicon tracking cameras and a tracking computer (Quad Core Intel Xeon 3.6 GHz processor, NVidia Quadro K2000 graphics card, 8GB RAM) running Windows. The head-mounted display and Apex hand-held device were tracked at a rate of 240 Hz using Tracker 2 (Vicon) software. The graphics computer (Linux) that generated the experimental stimulus had an 8 core AMD Opteron 6212 processor, dual NVidia GeForce GTX 590 graphics cards, 16GB RAM.

Experiments

In both VR and the real-world experiment, participants took as much time as they needed to view and memorize the box positions, although this was typically between 10 and 20 seconds. If they attempted to leave the start zone to walk closer to the target boxes, the whole scene disappeared in VR or the participant was inhibited by the experimenter in the real world. The indirect and direct walking conditions were intermingled and tested in a randomised order. The participants did not know which condition they were being tested in at the beginning of each trial while standing at the start zone. In VR, the pointing zone was indicated by a colored poster (colored according to the color of the box to which they should point) and this poster only appeared after they left the start zone. Similarly, in the real-world task, there were three white posters at the three pointing zones (see Fig. 1c) and the participant was only told which one to stop at after they had left the start zone. In VR, the poster changed its color after each shot to indicate the next target box to point at. In the real world, the experimenter told the participant which box to point at. At the end of each trial (when they returned to the Start zone to view a new box layout), the participant received a score ranging from 0–100% reflecting the accuracy of all the shots (as this increased participants' motivation) but this information could not be used to infer the direction or magnitude of their pointing error on any given shot.

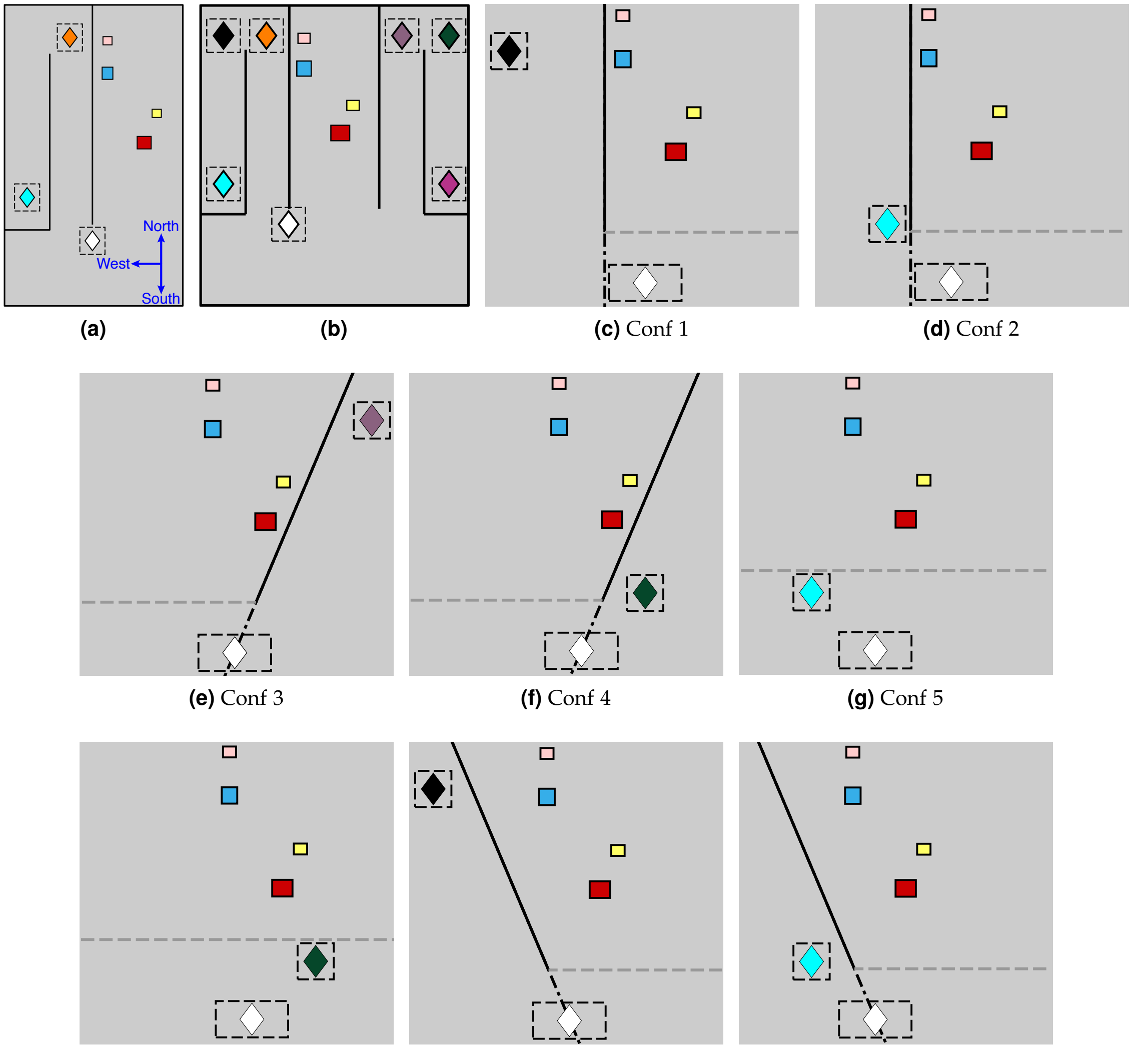

(a) **(b)** **(c)** Conf 1 **(d)** Conf 2

(e) Conf 3 **(f)** Conf 4 **(g)** Conf 5

(h) Conf 6 **(i)** Conf 7 **(j)** Conf 8

Figure S1. Plan view of (a) Experiment 2, (b), Experiment 3 and (c–j) Experiment 4 with 8 different configurations. Black solid line indicates walls that were always present. In (c–j), the gray dotted line shows the wall that appeared when the participant pressed a button at the start zone (white diamond). The black dotted line shows the wall that appeared after participant left the start zone. In (g) and (h) the wall remained in the same place.

Results

Individual Participant Data

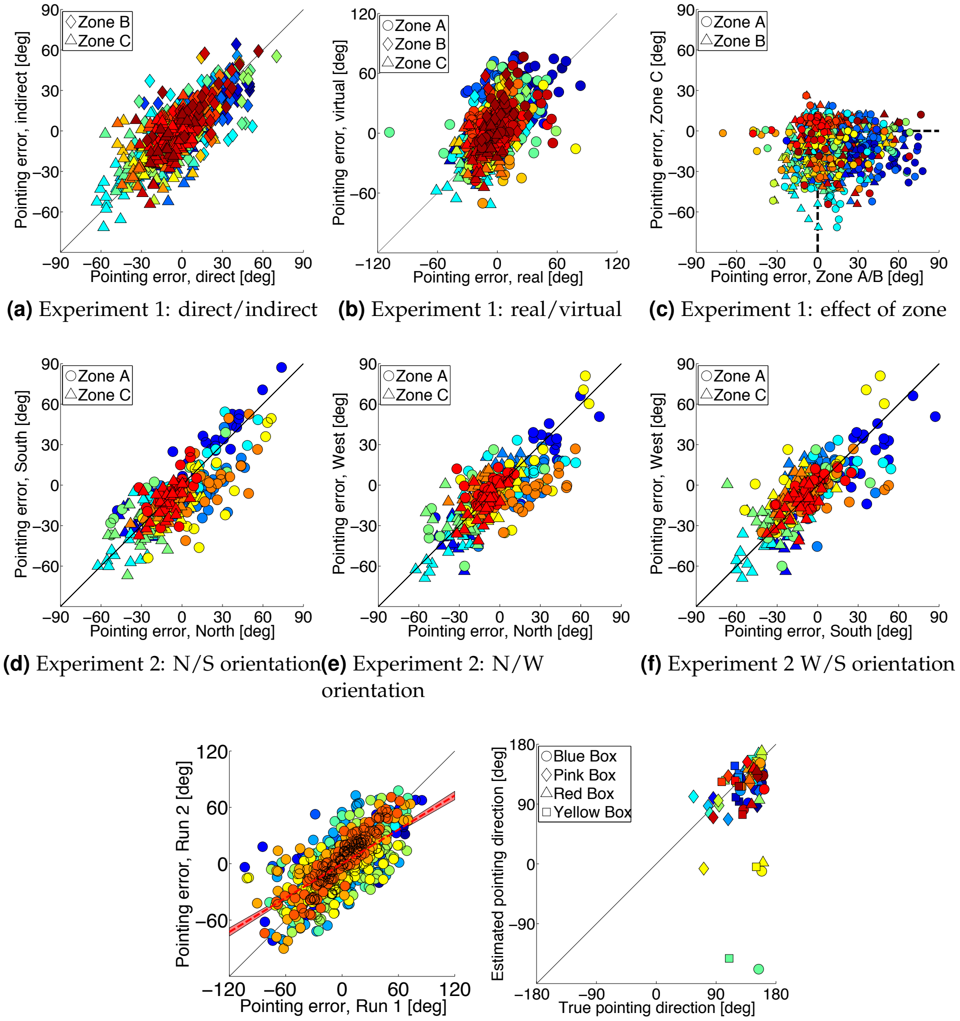

(a) Experiment 1: direct/indirect **(b)** Experiment 1: real/virtual **(c)** Experiment 1: effect of zone

(d) Experiment 2: N/S orientation **(e)** Experiment 2: N/W orientation **(f)** Experiment 2 W/S orientation

(g) Experiment 4: repeated trials **(h)** Experiment 1: very first trial

Figure S2. Individual participant data. Colors indicate different participants. (a) corresponds to Fig. 2 (direct versus indirect walking), (b) to Fig. 3a (real versus VR), (c) corresponds to Fig. 3c (zone C versus zone A/B) and (d) corresponds to Fig. 3b ('North'-facing versus 'South'-facing). (e) and (f) show data from an additional condition (participants initially faced 'West') compared to the 'North' and 'South' conditions. (g) In Experiment 4, the same conditions were repeated twice; pointing errors for the two runs are plotted against each other (slope is 0.61, indicating a smaller range of errors on the second run). (f) The very first time that participants viewed the stimulus in the real world, they were given no instructions at the start zone. Then, at the pointing zone, they were asked to point to the four boxes in a random order, eight times each (i.e. 32 shots per participant). It is debatable whether a post-hoc power analysis is of value in this instance but, for the record, this shows that the power achieved to rule out the correlation shown in Fig. 2d/Fig. S2a occurring by chance is, to a very close approximation, 100%. More relevantly, the same pattern of biases is found throughout the remaining experiments in the paper.

Alternative Definitions of Pointing Direction

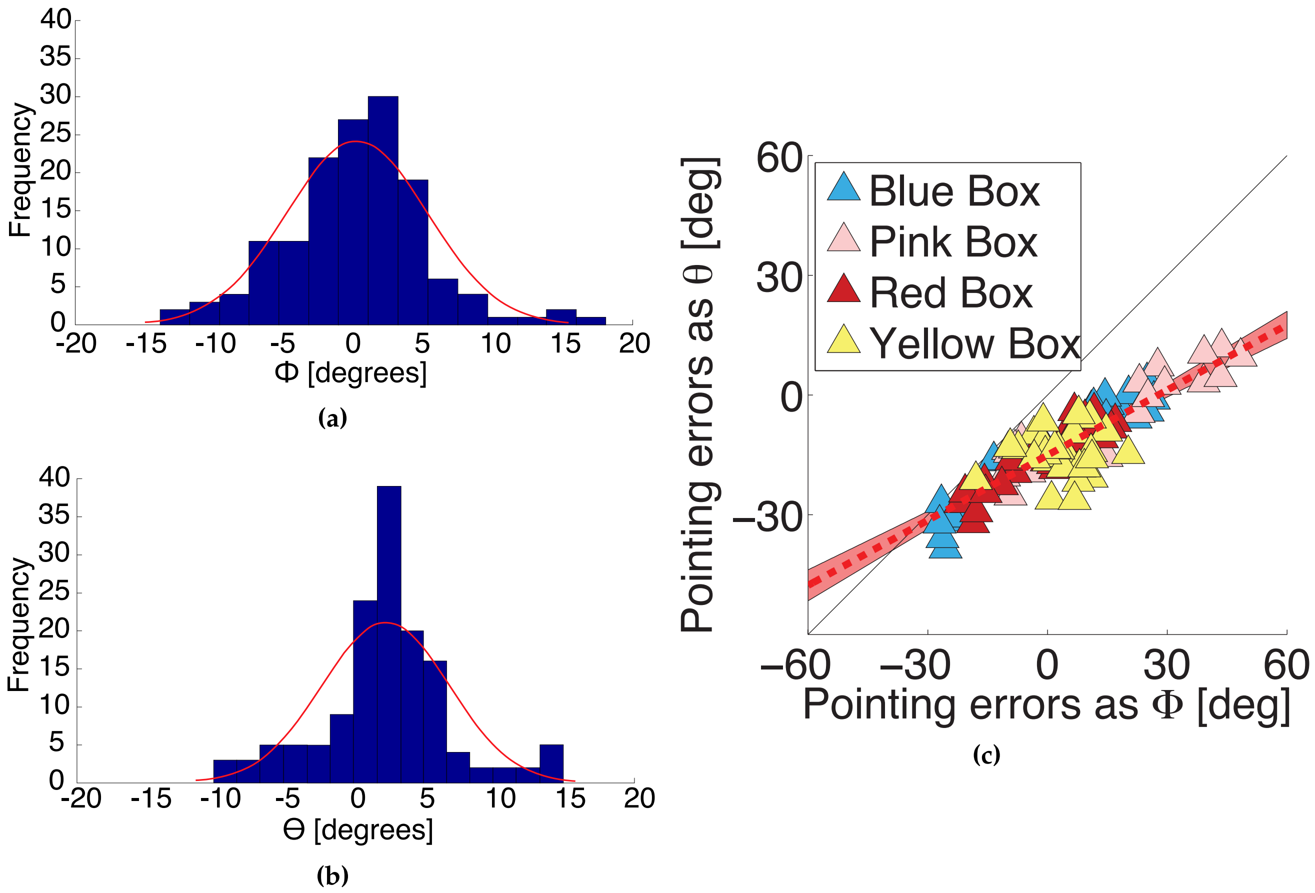

Figure S3. Evidence supporting the choice of 'shooting' angle (ϕ) rather than visual direction (θ) as the most appropriate definition of pointing direction. In a control condition, participants pointed at target boxes from the start zone so the target was visible (18 participants tested on 2 box layouts with 4 target boxes in each layout, 144 shots in total). Their instruction was to 'shoot at the box', just as it was in the spatial updating experiments. (a) Distribution of pointing errors when target direction is defined relative to the pointing device and shooting direction is defined by the orientation of the device (see ϕ in Fig. 1d). (b) As for (a) but with pointing errors defined as the difference in visual direction of the target and the pointing device as measured from the cyclopean point (see θ in Fig. 1e). The mean of the distribution is significantly biased for θ (t-test, $p < 0.001$) but not for ϕ ($p = 0.605$) suggesting ϕ reflects participants' intentions when pointing. (c) There is a significant correlation between the ϕ and θ measures in the spatial updating experiments (correlation coefficient 0.91). These data are from Experiment 1. When $\phi = 0$, θ is about $-20°$. A negative bias is the direction of bias that would be created if a right-handed participant held the pointing device slightly out to their right and pointed to a target directly ahead of them.

Visual Models

Noisy-path-integration Model

The noisy-path-integration model simulates a moving observer storing an egocentric map of the box positions by constantly updating the heading direction with respect to 'North' (α) and estimating the distance traveled on each step (d). Here, we assume that the observer misestimates α and d with a constant error (multiplicative calibration errors ω_α and ω_d respectively). This leads to a cumulative error in the estimate of the participant's location. The box locations are assumed to be known correctly.

Initially, the position of the boxes is given, by definition, as follows, where S_0^b is the starting distance of box b and angle η_0^b is its visual direction with respect to 'North' ($\frac{\pi}{2}$) as viewed from the start position (boxes are still

visible here):

$$\eta_0^b = \frac{\pi}{2} - \text{atan2}(boxpos_x^b - pos_{0,x}, boxpos_y^b - pos_{0,y})$$
$$S_0^b = \sqrt{(boxpos_x^b - pos_{0,x})^2 + (boxpos_y^b - pos_{0,y})^2}$$

with $boxpos_x^b$ and $boxpos_y^b$ being the x- and y-coordinates of the bth box, $pos_{0,x}$ and $pos_{0,y}$ the cyclopean point of the observer at the start position. At subsequent steps, when the boxes become obscured by the wall, the polar coordinates are calculated using the following equations:

$$\eta_n^b = \arctan\left(\frac{b}{a}\right)$$
$$S_n^b = \sqrt{a^2 + b^2}$$

with

$$a = \sin(\eta_{n-1}^b) \cdot S_{n-1}^b - \sin(\alpha \cdot \omega_\alpha) \cdot (d \cdot \omega_d)$$
$$b = \cos(\eta_{n-1}^b) \cdot S_{n-1}^b - \cos(\alpha \cdot \omega_\alpha) \cdot (d \cdot \omega_d).$$

We fitted this model to all the data from all participants in Experiment 1 by varying the two free parameters, ω_α and ω_d, to give the minimum root-mean-square-error between the actual and predicted pointing directions. (see Fig. S4a).

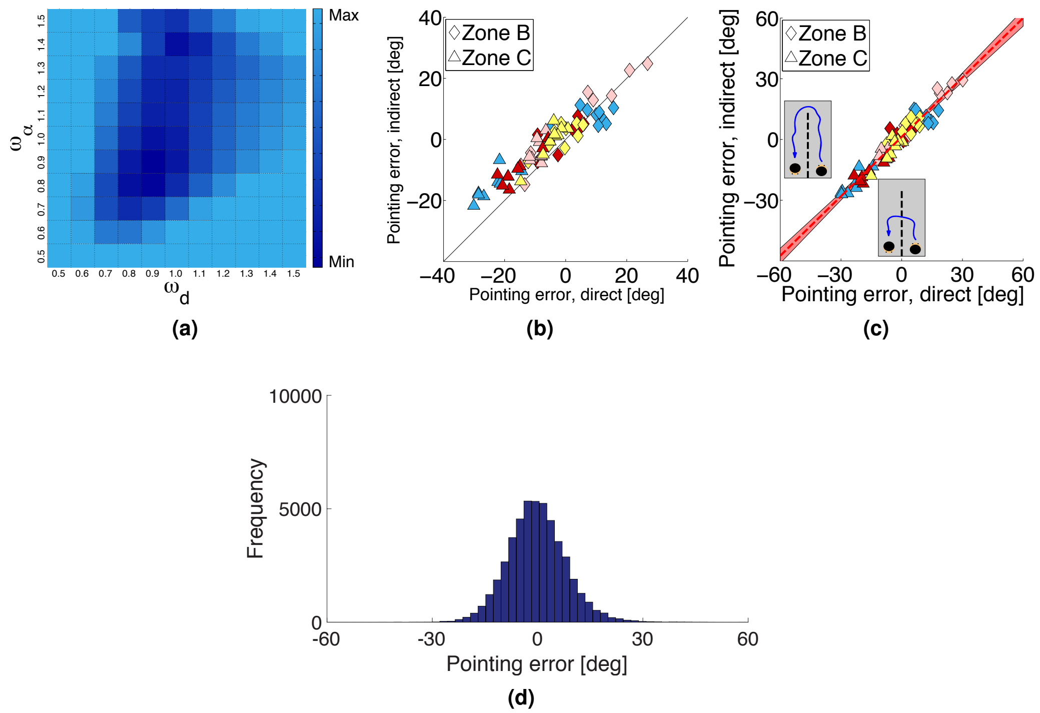

Figure S4. Illustration of the noisy-path-integration model. (a) Using the data from all participants, this plots shows the different values of the root-mean-square error (RMSE) between data and model measured in degrees using different combinations of the model parameters ω_d (distance bias) and ω_α (angular bias). Any combination with an RMSE larger than twice that of the ground truth model for the indirect condition of Experiment 1 ($RMSE_{lim} = 2 \cdot 16.6^\circ$) has been set to that value ('Max'). Dark blue squares show the calibration errors with lower RMSE values, the lowest is at $(0.9, 0.9)$ with an RMSE value of 13.2° ('Min'). (b) Comparison of noisy-path-integration predictions for direct and indirect walking. Error predicted by the noisy-path-integration model for the indirect walking condition plotted against this value for direct walking trails. As in Fig. 2d, each symbol is based on the mean data for 20 participants. The data for zone C (triangles) is most informative as the length difference between the direct and indirect paths is most extreme in this case. Here, the errors for indirect walking are significantly more positive than the errors for direct walking (direct walking $M = 6.06, SD = 3.19$, indirect walking $M = 0.764, SD = 2.76$, $t(35) = 18.5, p < 0.001$), whereas the experimental data for these two conditions, reproduced here in (c) from Fig. 2d, were not significantly different. (d) Histogram of prediction errors calculated 100 times for each box in each layout, using the walking trajectory of every participant tested in the indirect walking condition of Experiment 1 at one of the pointing zones (zone C) with a normally distributed random noise on the estimate of η_n^b. The mean of the pointing errors is not significantly different from zero. The mean of the distribution for zone A and zone B was also not significantly different from zero.

Zero-mean Noise

If, instead, we assume that the noisy-path-integration noise has zero mean then there is no systematic effect on pointing, which we demonstrate as follows for an estimate of orientation. We added a normally distributed random error to the estimate of visual direction with respect to 'North' on each step, η_n^b:

$$\begin{aligned} \eta_n^b &= \arctan\left(\frac{b}{a}\right) + E \\ E &= randn\left(0, \frac{\pi}{360}\right) \end{aligned} \tag{1}$$

with the function $randn(\mu, \sigma)$ returning a random number drawn from a distribution with a standard deviation σ, and a mean μ. E is a random error added to the estimate of η_n^b, drawn from a distribution with a standard

deviation of $\sigma = \frac{\pi}{360}$ radians, and a mean of $\mu = 0$.

Using Eq. (1), predictions of pointing directions were calculated with a random additive noise on the direction of 'North'. Calculating the directions 100 times for each box in each layout, using the walking trajectory of every participant tested in the indirect walking condition of *Experiment 1* at pointing Zone C, a histogram of errors is plotted in Fig. S4d (and the same result applies for Zone A and Zone B).

Abathic Model

Johnston[1] shows psychophysical data described by a linear relationship between estimated (or 'scaling') distance to physical viewing distance (e.g. their Fig. 7). In general, we can fit the two parameters (intercept and slope) to our pointing data (Fig. 7a). In our case, the best-fitting values are a slope of 1.03 and an intercept of 0.17, which corresponds to an abathic distance of -5.66. Specifically, the misestimated egocentric distances of the boxes, d^b_{est} is:

$$d^b_{est} = d^b_{true} * 1.03 + 0.17 \tag{2}$$

where d^b_{true} is the true egocentric distance and $b = [1, \ldots, 4]$ the index for each box.

Retrofit Model

We can allow box position to vary and calculate the maximum likelihood configuration of boxes that could account for the participant data (separately for each Experiment). We considered a 200 by 200 grid of possible box positions centered on the true box locations. For each possible box position the likelihood of the participant representing the box as being at that location (given their pointing responses from 3 different zones) can be defined as:

$$L^{b,l,k}_{p,m} = \frac{1}{\sigma \cdot \sqrt{2 \cdot \pi}} \cdot e^{-\frac{(a_k - \mu)^2}{2 \cdot \sigma^2}} \tag{3}$$

with $p = [1, \ldots, 20]$ for 20 participants, and $b = [1, \ldots, 4]$ for all 4 boxes in $l = [1, \ldots, 9]$ 9 box layouts, and $m = [1, \ldots, 3]$ for all 3 testing zones, and $k = [1, \ldots, 4000]$ for all the possible box positions and a_k is the angular error between the estimated pointing direction and the kth possible box position. We assume no systematic bias (μ = 0) and an arbitrarily chosen σ = 15. The maximum likelihood is then calculated for each box in each layout across all participants across all zones:

$$L^{b,l,k} = L^{b,l,k}_{1,1} \cdot L^{b,l,k}_{1,2} \cdot L^{b,l,k}_{1,3} \cdot L^{b,l,k}_{2,1} \cdot \ldots \cdot L^{b,l,k}_{P,M} \tag{4}$$

where $P = 20$ for the total number of participants and $M = 3$ for the total number of shooting zones.

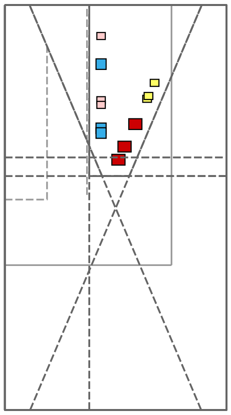

(a) Experiment 1, 2, and 4

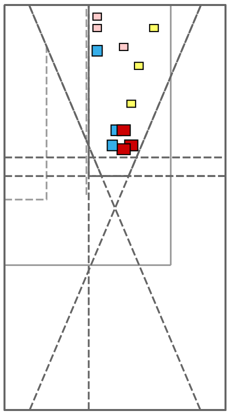

(b) Experiment 1, 2, and 4

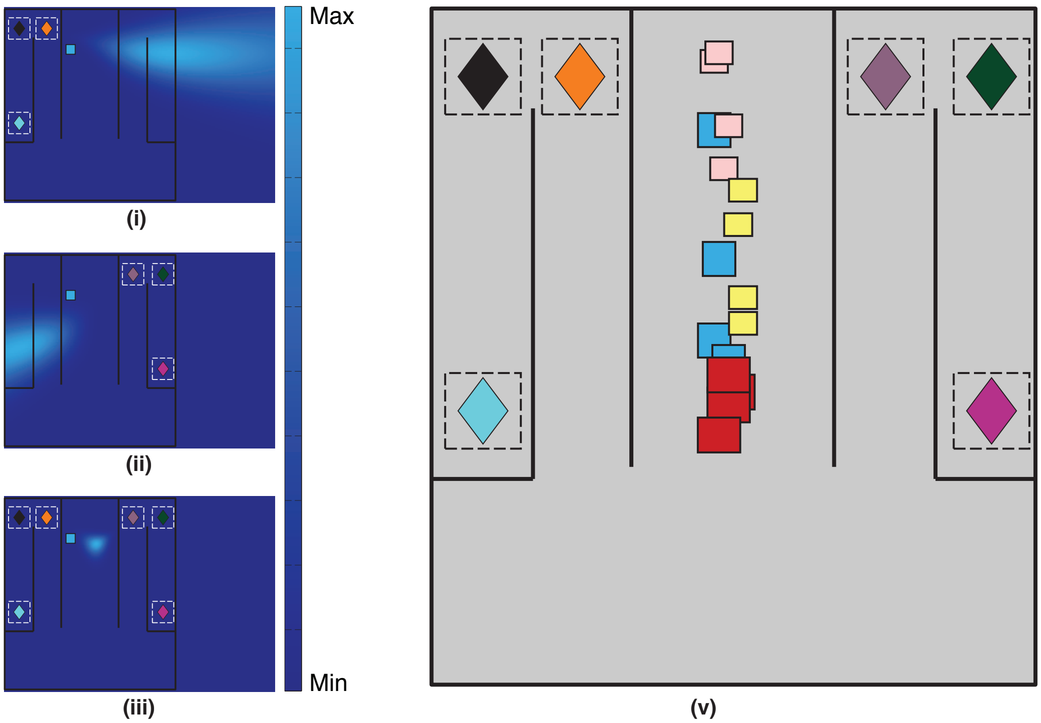

(c) Experiment 3

Figure S5. (a–b) Box layouts tested in Experiment 1, 2, and 4: (a) Real box positions of 3 box layouts plotted in the same plan view. (b) Predicted box layouts calculated using the 'retrofit' model, based on combined data from all studies. (c) Experiment 3, 'retrofit' model using the estimated directions of one participant pointing to the blue box in one layout (i) at zones A–C alone or (ii) at zones D–F alone or (iii) A–F all together. (v) Similar to (iii) but now showing the maximum likelihood location for all the boxes in all layouts. In both (iii) and (v), the predicted box locations are shifted towards a 'North-South' plane in the center of the room.

Model Comparison

Per-participant comparison between path-integration, abathic distortion and 'projection plane' models in Experiment 1

Participant	Model residuals (RMSE) [Degrees]		
	Noisy path	Abathic distance	Projection plane
P1	16.11	11.55	7.65
P2	27.54	21.06	18.73
P3	11.68	10.33	7.89
P4	13.92	13.34	10.93
P5	23.74	19.28	17.57
P6	8.66	8.43	11.21
P7	12.66	12.97	11.68
P8	22.56	23.92	19.76
P9	14.56	12.98	9.57
P10	21.16	21.39	14.45
P11	18.15	19.61	15.72
P12	16.23	9.36	17.80
P13	14.85	16.79	11.87
P14	16.78	14.36	10.66
P15	15.75	10.09	13.15
P16	11.27	12.57	10.46
P17	13.09	12.05	9.69
P18	13.65	14.74	10.31
P19	15.74	16.40	11.11
P20	15.47	13.75	11.12

Table S1. Root-mean-square residuals (RMSE) of the pointing data relative to two models of pointing error for Experiment 1. RMSE values are shown per participant for two standard models and the projection plane model. See Sections Noisy-path-integration Model and Abathic Model for details of the noisy-path-integration and abathic models respectively. Gray-colored cells show lowest RMSE out of the 3 different models for each participant. Overall, the RMSE for the projection-plane model is 13.0, for the noisy-path-integration model is 16.8 and for the abathic-distance-distortion model is 15.3 degrees. One participant had knowledge of the type of hypothesis that was being explored in the experiments (P15). Their RMSE values in Experiment 1 shown here and for the values shown in Fig. 5 and Fig. 6 were within the range for other participants.

Model Comparisons using Two Model Selection Criteria, AIC and BIC

**Experiment**	1		2		3		4	
**Criteria**	Projection	Retrofit	Projection	Retrofit	Projection	Retrofit	Projection	Retrofit
AIC	156.7	258.5	362.2	509.5	460.7	446.4	961.7	1177.7
BIC	158.3	298.1	364.5	566.4	463.3	510.5	964.9	1259.1
# of Parameters	0	24	0	24	0	24	0	24

Table S2. A comparison of the fits of the projection plane model and the retrofit model using Akaike (AIC) and Bayesian (BIC) Information Criteria which penalize a model according to the number of free parameters it has. Lower values indicate a better fit.

References

1. Johnston, E. B. Systematic distortions of shape from steoreposis. *Vis. Res.* **31**, 1351–1360 (1991).
